# Food Waste Biotransformation into Food Ingredients: A Brief Overview of Challenges and Opportunities

**DOI:** 10.3390/foods13213389

**Published:** 2024-10-24

**Authors:** José Pinela, Mikel Añibarro-Ortega, Lillian Barros

**Affiliations:** 1 Centro de Investigação de Montanha (CIMO), Instituto Politécnico de Bragança, Campus de Santa Apolónia, 5300-253 Bragança, Portugal; mikel@ipb.pt (M.A.-O.); lillian@ipb.pt (L.B.); 2 Laboratório Associado para a Sustentabilidade e Tecnologia em Regiões de Montanha (SusTEC), Instituto Politécnico de Bragança, Campus de Santa Apolónia, 5300-253 Bragança, Portugal; 3 National Institute for Agricultural and Veterinary Research (INIAV), I.P., Rua dos Lágidos, Lugar da Madalena, 4485-655 Vairão, Vila do Conde, Portugal; 4 Nutrition and Bromatology Group, Department of Analytical and Food Chemistry, Faculty of Food Science and Technology, University of Vigo, Ourense Campus, E-32004 Ourense, Spain

**Keywords:** food sustainability, bio-based ingredients, natural food additives, lignocellulosic biomass, bioconversion, fermentation, sustainable development

## Abstract

In today’s global context, challenges persist in preventing agri-food waste due to factors like limited consumer awareness and improper food-handling practices throughout the entire farm-to-fork continuum. Introducing a forward-thinking solution, the upcycling of renewable feedstock materials (i.e., agri-food waste and by-products) into value-added ingredients presents an opportunity for a more sustainable and circular food value chain. While multi-product cascade biorefining schemes show promise due to their greater techno-economic viability, several biotechnological hurdles remain to be overcome at many levels. This mini-review provides a succinct overview of the biotechnological and societal challenges requiring attention while highlighting valuable food-grade compounds derived from biotransformation processes. These bio-based ingredients include organic acids, phenolic compounds, bioactive peptides, and sugars and offer diverse applications as antioxidants, preservatives, flavorings, sweeteners, or prebiotics in foodstuffs and other consumer goods. Therefore, these upcycled products emerge as a sustainable alternative to certain potentially harmful artificial food additives that are still in use or have already been banned from the industry.

## 1. Introduction

Nowadays, nearly one-third of all food produced around the globe is lost or wasted each year throughout the entire food life cycle, which otherwise would be enough to feed two billion people, more than twice the number of undernourished people worldwide [1]. Food loss and waste (FLW) result from various causes and represent an economic, social, and environmental problem that has been intensified by the scarcity of natural resources, lack of arable land, and climate change. Although the terms “food loss” and “food waste” are often used interchangeably, it is important to distinguish between them and the circumstances of their occurrence, especially when identifying causes and developing solutions to address the issue. According to the Food and Agriculture Organization (FAO) of the United Nations (ON), food loss refers to reductions in the quantity or quality of food that occur during the harvesting and agricultural production activities (including storage, transport, and processing), while food waste occurs at the manufacturing, retailing, and household consumption stages [2,3]. Therefore, to achieve sustainable production and consumption patterns, as envisioned by the Sustainable Development Goal 12 of the UN’s 2030 Agenda for Sustainable Development [4], it will be critical to adopt behavioral approaches and technologies for food loss reduction and food waste prevention and their cost-effective valorization. For simplicity, the term FLW is used throughout this review.

The increase in global food supply is expected to maintain significant FLW generation [5], which must be addressed from both a managerial and technological perspective [6]. Current in-practice operations for FLW treatment include landfilling, composting, incineration, or, at best, its use for animal feed, biofertilizer, and energy production [6,7]; fortunately, alternative biotechnological and solid–liquid extraction processes can offer better environmental and techno-economic performance [7,8]. The global concern caused by the mismanagement of FLW can thus be mitigated by innovative technologies that open new horizons for their bioconversion into high-value-added products in a circular loop. Its sub-zero decomposition by biorefineries manufactures waste into bio-based products such as specialty chemicals for the food, nutraceutical, biotechnological, and agricultural industries, among other sectors, while reducing the use of fossil resources and supporting the transition to a circular bioeconomy at lower virgin material intensity. This approach may help prevent hunger, food security issues, and global warming. However, FLW conversion is fraught with challenges that require multiple steps to achieve high efficiency in transforming these materials into valuable products. Although biotechnological, chemical, thermochemical, and physical processes are available for biomass conversion and valorization, their efficiency can be very low due to the need for feedstock pretreatments or due to the early-stage maturity and readiness level of such technologies.

This short review aims to discuss the potential of FLW for producing food-grade ingredients through multi-product cascade biorefinery processes, highlighting the main challenges and opportunities. This forward-thinking approach addresses a growing scientific challenge with the potential to enhance the utilization and circularity of low-value feedstock. The food-grade ingredients produced through these methods could serve as eco-sustainable alternatives to some synthetic or artificially obtained substances currently used as additives in the food industry and other sectors. So, could FLW be a fresh start?

## 2. Challenges and Opportunities to FLW Upcycling

The concept of renewable natural resources is evolving thanks to the emergence of integrated biorefinery structures aimed at co-producing various bio-based products [9]. Indeed, single bioprocess or product-based approaches have several techno-economic limitations, while integrated strategies comprising multiple cascading biotechnological processes to recover as many valuable FLW constituents as possible promote waste utilization, economic viability, and sustainability [6,9]. Furthermore, it is imperative to consider the trade-offs between profitability and environmental impacts.

**Figure 1:**
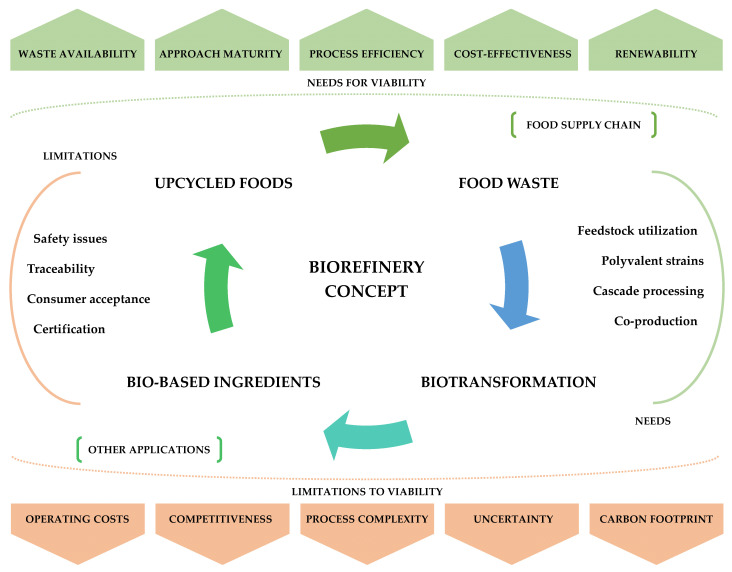
Simplified overview of the biorefinery concept for biotransformation of FWL into bio-based ingredients. FLW from the food supply chain can be used as feedstock to produce food-grade ingredients. Biorefineries must be prepared to handle multiple feedstocks and support cascade co-production processes. Polyvalent microbial strains may be required to ensure efficient bioprocessing. The resulting bio-based ingredients can be incorporated into upcycled foods (or other consumer goods). Still, possible limitations related to food safety, traceability, certification, and consumer acceptance can be downstream barriers. The upcycled foods can generate new waste that can also be recovered to close the loop. The green boxes highlight that adequate and consistent feedstock availability, high technological maturity, process efficiency, and cost-effectiveness are requirements for biorefinery viability. In contrast, factors such as high operating costs, process complexity, competitiveness, uncertainty, and carbon footprint (highlighted in brown) must be minimized.

As illustrated in Figure 1, several prerequisites are essential for the viability of biorefineries, including the consistent availability of suitable feedstock, high technology readiness level, and efficiency and cost-effective processes. Additionally, FLW biorefineries should be capable of handling multiple feedstocks to address potential scenarios of growing scarcity in specific resources. On the other hand, factors such as operating costs, process complexity, competitiveness, uncertainty, and carbon footprint must be minimized to ensure success [10,11]. A crucial aspect of this assessment is determining whether the production process is less resource-intensive than manufacturing the same product from virgin materials while also comparing alternative valorization pathways. Figure 1 further highlights the importance of cascade co-production approaches during biotransformation, which may require the utilization of polyvalent microbial strains with superior metabolizing capacity to enhance product yields. In addition, the market price of value-added products plays a pivotal role in determining the biorefinery’s profitability, highlighting the need for thorough market analysis before implementation. Economies of scale are another important factor as they can greatly influence profitability. However, not all FLW feedstocks offer the same potential. The most profitable scenarios typically involve centralized production with fewer plants, allowing for economies of scale [11]. At the same time, optimizing feedstock availability and transportation must be systematically addressed to avoid trade-offs, such as increased environmental impacts and logistical externalities, which could undermine the profitability of FLW biorefineries. Challenges related to the safety, traceability, certification, and consumer acceptance of upcycled ingredients and food products may also arise downstream in the value chain.

The food supply chain generates a substantial amount of FLW biomass with variable composition and physicochemical properties, sourced from diverse origins such as industrial processing, marketplaces, restaurants, and households [12]. In 2022, around 1.05 billion tons of food waste were generated globally, 60% of which came from households, 28% from food services, and 12% from retail [13]. While FLW from the consumption stage is less affected by seasonal availability, it is generally heterogeneous and less exploited by biorefineries. In contrast, FLW from food processing and manufacturing stages is usually seasonality dependent but tends to have a greater compositional homogeneity (e.g., straw, husks, bagasse, pomace) because they are sourced from specific industries or value chains with predictable output patterns, allowing for targeted collection and the possibility of source separation to minimize contamination. As a result, this makes this FLW more suitable as feedstock for biorefineries to produce high-value-added products and chemicals [10]. However, FLW biorefineries face complex challenges due to the variability and heterogeneity of biomass compared to dedicated feedstock. These facility schemes should be designed to switch between seasonal feedstocks and utilize mixed supplies rather than a single source. Although the seasonal flow of feedstock can be managed through airtight storage and preservation techniques, a combinatorial problem-solving approach is necessary to handle all these challenges effectively.

**Figure 2:**
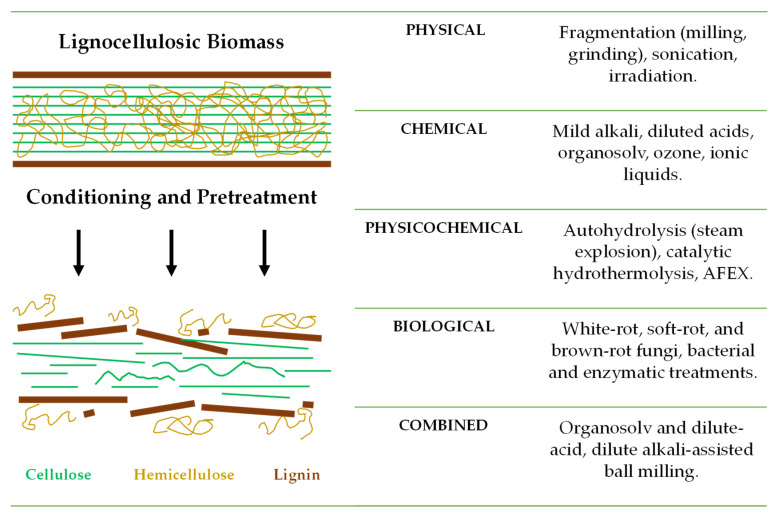
Representative structure of lignocellulosic biomass and conditioning and pretreatment methods used to improve its fractionation into cellulose, hemicellulose, and lignin. Physical methods (e.g., milling, grinding, sonication, irradiation) increase surface area and mass transfer, while chemical (e.g., mild alkali, diluted acid, organosolv, ozone, ionic liquids), physicochemical (e.g., autohydrolysis/steam explosion, catalytic hydrothermolysis, ammonia fiber expansion (AFEX)), biological (e.g., white-rot, soft-rot, and brown-rot fungi, bacteria, enzymes), and combined (e.g., organosolv and dilute-acid, dilute-alkali-assisted ball milling) pretreatments generate hydrolysates.

In addition to phenolic compounds, carotenoids, and many other phytoconstituents, plant-derived FLW contains carbohydrates that serve as high-potential carbon sources for microbial fermentation. While simple sugars are the most suitable substrates, they are not abundantly present in plant-derived FLW. On the other hand, polysaccharides such as cellulose, hemicellulose, and lignin are found in adequate concentrations and can be converted into their monomeric fermentable units. In lignocellulosic biomass, cellulose typically represents the most considerable fraction (30–50%), followed by hemicellulose (20–35%) and lignin (15–30%) [14]. However, cellulose is embedded in and linked to hemicellulose and lignin (Figure 2), creating a significant challenge for enzymes to convert it directly into monosaccharides. The recalcitrance of the cell wall, mainly due to the presence of lignin and lignin–carbohydrate complexes, is, therefore, a significant barrier to efficient cellulose conversion [15].

**Figure 3:**
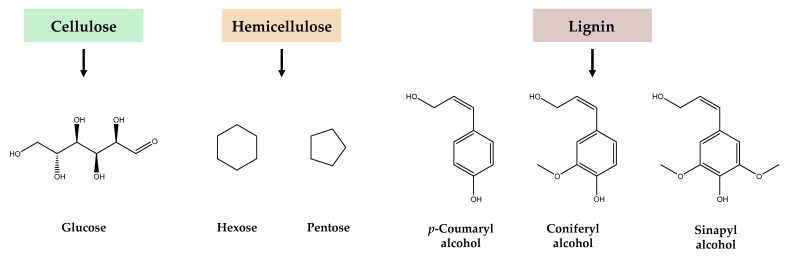
Carbon sources derived from cellulose (glucose), hemicellulose (mannose, galactose, rhamnose, arabinose, xylose), and lignin (*p*-coumaryl alcohol, coniferyl alcohol, sinapyl alcohol) after fractionation of lignocellulosic biomass.

To overcome this recalcitrance, biomass pretreatments are necessary to solubilize lignin and hemicellulose, break down the structural complexity of these complex polymers, and facilitate subsequent hydrolysis (Figure 2). Depending on the biomass characteristics, initial conditioning steps such as milling, grinding, and sonication may be required to increase the surface area for reactions and enhance mass transfer. Additional steps like washing to remove dirt and pigment matter, as well as drying, may also be required. Following conditioning, chemical (e.g., mild alkali, diluted acid, organosolv, and ozone), physicochemical (e.g., autohydrolysis, hydrothermolysis, and ammonia fiber expansion (AFEX)), biological (e.g., white-rot, soft-rot, and brown-rot fungi, bacteria, and enzymes), or combined pretreatments (Figure 2) can be applied to hydrolyze the lignocellulosic biomass [6,9,14]. These pretreatments generate different carbon sources as FLW hydrolysates (Figure 3) to improve the yield and efficiency of subsequent microbial fermentation and biotransformation processes. Among the pretreatment methods, mild alkali is particularly popular because it effectively removes a significant portion of lignin and hemicellulose, increasing cellulose accessibility and subsequent bioconversion into valuable products. Among the green methods, organosolv stands out for its ability to selectively dissolve lignin and hemicellulose while leaving cellulose intact. This emerging approach uses organic solvents such as ethanol, methanol, acetone, and ethylene glycol that can be recovered and reused, making it an environmentally sustainable option for lignocellulose biomass fractionation [15]. After biomass conditioning and pretreatment, additional purification steps, such as filtration, washing, and centrifugation, or bleaching with hypochlorite or peroxide, may be required to remove residual lignin and hemicellulose [15].

In the case of nitrogen-rich FLW biomass, it can originate peptides and amino acids essential for microbial growth [16], e.g., soybean meal can provide the nitrogen needed for lactic acid production [17]. Therefore, targeting FLW biomass as a feedstock for biorefineries producing industrially important specialty chemicals such as food-grade organic acids, phenolic compounds, bioactive peptides, sweeteners, and prebiotics can be seen as a sustainable, future-oriented approach. Even so, its industrial exploitation has been limited, and there are few studies evaluating its economic viability at the industrial scale [10].

## 3. FLW-Derived Food Additives and Ingredients

Food additives are substances intentionally added to food for specific technological purposes but not consumed as food themselves. They are classified into various functional classes, including antioxidants, preservatives, flavorings, sweeteners, acidity regulators, and colors, among others [18]. Over the last decades, scientific studies have alerted to the potential allergenic and carcinogenic effects of certain artificial food additives [19,20], prompting the industry to gradually phase them out [20]. For example, the European Union has banned substances like titanium dioxide (E171), used as a white colorant [21], and potassium bromate (E924), used as a flour improver [22], after the European Food Safety Authority (EFSA) considered them no longer safe as food additives. Potassium bromate is still permitted in the United States at a maximum permissible concentration of 0.02 mg/kg in baked goods [23]. However, in 2023, California emitted an assembly bill banning potassium bromate, along with other additives such as red no. 3 or erythrosine (E127) [24]. This approved act, set to take effect in 2027, marked the first time a state has banned a Food and Drug Administration (FDA)-approved food additive, highlighting the ongoing debate and uncertainty surrounding food additives. While competent authorities regularly review the toxicity of food additives and real population exposures, some assessments are over a decade old, indicating that new evidence will soon be needed.

Consumers today are much more interested in how their food is produced and what ingredients it contains [25]. As a result, they are favoring clean-labeled concepts that emphasize simplicity, transparency, and natural origins [25,26]. This shift in consumer preference is driven by a growing awareness of the health and environmental impacts of artificial food additives and ultra-processed ingredients [27]. Consequently, the food industry has been prompted to reduce or eliminate some artificial substances or to replace them with natural alternatives sourced mostly from plants but also from microorganisms and animals [28]. However, this can be challenging due to the limited availability, stability, or higher cost of natural substitutes [27]. In response to these market trends, alongside the growing need to valorize FLW biomass, there has been a push to develop bio-based, food-grade ingredients and additives. These innovations offer a wider range of natural counterparts compared to those produced artificially or from virgin materials. Additionally, rare sugars with potential health benefits can be obtained through these processes [29]. Bio-based food additives and ingredients derived from solid and liquid FLW via biotransformation processes are discussed below.

### 3.1. Organic Acids

Malic (E296), ascorbic (E300), lactic (E270), citric (E330), and succinic (E363) acids have been used as antioxidants, preservatives, acidulants, or flavoring agents in different foodstuffs and can be obtained from FLW [30,31,32], mainly through microbial fermentation [33,34]. Some of these compounds, such as lactic, succinic, and citric acids, are also promising building-block chemicals for other consumer goods [35,36,37]. *Aspergillus niger* can produce citric acid in submerged (SF) and solid-state (SSF) fermentation on fruit waste such as citrus and apple pomace and waste mixtures [9,34,38,39]. SSF is simpler and requires lower operating costs and downstream processing efforts than SF; despite allowing the use of FLW without pretreatment, it does not allow an efficient use of nutrients and is more difficult to scale up [40]. In turn, SF allows for more efficient process control and better use of substrates [40]. However, both methods require the subsequent recovery of citric acid from the fermented broth, which can be achieved by precipitation, extraction, or purification steps [40].

Regarding succinic acid, it can be produced from starch-rich and mixed FLW feedstock such as orange peel, wheat bran, and bakery and restaurant waste, using *Actinobacillus succinogenes*, *Anaerobiospirillum succiniciproducens*, *Mannheimia succiniciproducens*, and recombinant *Escherichia coli* strains as fermentative bacteria [35,36]. Theoretically, two molecules of succinic acid can be produced from one monosaccharide molecule. In addition, succinic acid fermentation can occur anaerobically with carbon dioxide consumption [35,36]. For wheat-based materials, the fermentative feedstock can be made by SSF with *Aspergillus awamori* and *A. oryzae*, which produce enzyme complexes rich in amylolytic and proteolytic enzymes, respectively. In turn, during succinic acid production, MgCO_3_ can be supplemented as a neutralizing agent to control the pH and improve the process yield, since this variable prevents cell flocculation and prolongs the stationary phase [36].

Lactic acid is another important organic acid in the food industry, as it is a preservative and flavoring agent. It can be produced by lactic acid bacteria (LAB) such as *Lactobacillus* spp. through sugar fermentation [41]. Mixed waste from supermarkets, restaurants, and kitchens, among other FLW, can provide the nitrogen needed for LAB cultivation; however, as shown in Figure 2, lignocellulosic materials need pretreatment due to their recalcitrant nature [36,42]. Industrial operations can yield about 90% of calcium lactate based on the glucose fed, which is neutralized to give lactic acid while producing ~1 ton of calcium sulfate per ton of lactic acid. Alternatively, the neutralization step could be eliminated by using separation and purification methods based on desalting and water-splitting electrodialysis [26]. Furthermore, engineered yeasts such as *Pichia stipitis* can ferment xylose to lactate, offering the possibility of converting all lignocellulosic sugars to this chemical and carrying out fermentation at a lower pH, thus excluding the need for neutralization [26].

### 3.2. Phenolic Compounds

Phenolic compounds, as plant secondary metabolites, are widely found in plant-based FLW [43]. Its interest comes from the wide range of bioactive and functional properties and health-promoting effects they display, thus having multiple applications in the food, pharmaceutical, and cosmetics sectors (e.g., benzoic acid, an antimicrobial compound commonly used as a food preservative, E210). Phenolic compounds in the free form are generally recovered using organic solvents and solid–liquid extraction methods assisted by different mass transfer intensification technologies [44,45]; however, these methods cannot extract those in the insoluble-bound form. For this purpose, microbial fermentation by SSF and SF has been applied to release bound phenolics in a more profitable and greener way than chemical-based hydrolysis, with SSF providing higher yields than SF [46,47]. Bioconversion of free phenolics and bioproduction of new bioactive compounds can also be achieved [48]. For example, SSF of pomegranate husk with *A. niger* can yield 8 kg of ellagic acid per ton of waste [49], and SSF of green coconut husk with *Phanerochaete chrysosporium* releases ferulic acid with its subsequent conversion into vanillin [50]. Enzymes such as α-amylase, laccase, β-glucosidase, tannin acyl hydrolase, and ellagitannin acyl hydrolase are involved in these bioprocesses [34]. As a drawback, some pathogenic microorganisms can grow on non-sterile substrates, representing a potential food safety hazard, and the co-production of unidentified substances can make the isolation of target compounds difficult [47], an issue that deserves further investigation. However, multi-product cascade biorefineries should be prioritized to recover phenolic compounds together with other high-value products, e.g., polyphenols, organic acids, pectin, enzymes, and biofuel that can be obtained from apple pomace [51,52]. Furthermore, the potential impact of phenolic ingredients on the sensory properties of foods should be considered, as they have a great structural diversity and the ability to interact with other food constituents [53].

### 3.3. Bioactive Peptides: Bacteriocins

Bioactive peptides are protein fragments with diverse applications in both the food and medical industries. These biomolecules have long been used in food preservation for their antioxidant and antimicrobial properties and can also be incorporated into active food packaging materials due to their generally high thermal stability [54]. Bioactive peptides can be produced by different processes, such as chemical or enzymatic hydrolysis, microbial fermentation, or using innovative techniques [55]. The growing interest in these peptides is also driven by concerns over the widespread and indiscriminate use of antibiotics, which has contributed to the rise of multidrug-resistant microorganisms [56].

Bacteriocins, a type of antimicrobial peptide produced by bacteria, were the first bioactive peptide to be isolated and characterized. Each bacterial strain has specific immunity to its own bacteriocin, making the bacteriocin effective only against other bacteria. This allows bacteriocins to help their originating bacteria survive by inactivating competing microorganisms [54]. Bacteriocins are commonly named based on the genus or species of the producer strain and can be categorized into three major classes: class I (small post-translationally modified peptides like nisin), class II (small, heat-stable, non-modified peptides such as pediocin), and class III (larger, heat-labile peptides over 10 kDa, such as lysostaphin) bacteriocins. These classes can further be subdivided [56].

Bacteriocins with broad-spectrum bactericidal or bacteriostatic effects can be produced by LAB from low-value substrates such as sugar beet pulp, corn stover, cheese whey, and mussel processing waste. The antimicrobial activity of these peptides varies as a function of their molecular weight and physicochemical features, which are affected by fermentation conditions [57]. Nisin is the most studied food-grade class I bacteriocin, and it (more specifically, nisin A) is currently authorized as a food preservative (E234) [58,59]. This FDA-approved lantibiotic is produced by microbial fermentation in the exponential growth phase of *Lactococcus lactis* strains, being the only bacteriocin produced at an industrial scale. This process also allows for the co-production of lactic acid [59]. However, the production costs are still high, and therefore, high nisin-production strains and low-cost fermentation substrates have been in the research spotlight. In addition, hydrolysis pretreatments or nitrogen supplementation may be necessary to ensure efficient nisin production using FLW as carbon sources [59,60]. For example, for sugar beet pulp, which is a good feedstock for nisin production due to its low lignin content, a pretreatment combining acidification with 2% sulfuric acid, 121 °C heating, and enzymatic hydrolysis is required to ensure a higher yield of fermentable sugars. For corn stover, a more severe pretreatment involving an autohydrolysis at 230 °C and 9 g water/g feedstock is required, as well as an enzymatic process at 48.5 °C for 72 h to convert glucans and hemicelluloses into monomeric units (yielding ~70% conversion). Downstream separation and purification of nisin are also necessary [61]. This process begins with rotary vacuum filtration followed by precipitation with 40% ammonium sulfate to separate nisin from co-products. The retained nisin is then washed with water and dried using a fluid bed dryer [59].

Pediocin is another important example of bacteriocins. It is produced by LAB such as *Pediococcus acidilactici* and *Pediococcus pentosaceus* and exhibits strong activity against Gram-positive bacteria such as *Listeria monocytogenes*. Among the various pediocins, pediocin AcH 1 was the first to be studied in detail and characterized. Typically, microbial cultures of *P. acidilactici* and *P. pentosaceus* occur in aerobic conditions, which enhances the yield of pediocin production [62]. For example, this bacteriocin, along with lactic acid and crude protein, can be obtained using mussel processing wastes as a carbon source through enzymatic pre-treatment and batch fermentation, providing economic and environmental benefits [62]. Although pure pediocin is still unavailable on the market, efforts have been made to obtain a purified form of pediocin for food applications. Other antibacterial bacteriocins with potential application as food preservatives include enterocin and bovicin produced by *Enterococcus* and *Streptococcus* species, respectively [63,64]. More recently, glycocins or glycoactive bacteriocins, such as glycocin F, sublancin, thurandacin, bacillin, geocillicin, and enterocin 96, have been described with notable antimicrobial activity and a unique mechanism of action [65]. Although these bioactive peptides are not currently approved for commercial use, they could serve as natural clean-label alternatives to conventional preservatives to help reduce the risk of foodborne outbreaks [65].

### 3.4. Sweeteners and Prebiotics

Carbohydrate-rich FLW can be biotransformed into sweeteners and prebiotics such as sorbitol, mannitol (from mannose), xylitol, arabinitol, and rare sugars like galactooligosaccharides (GOS), xylooligosaccharides (XOS), and D-tagatose. These are promising substitutes for high-calorie sugars and artificial sweeteners and find multiple applications in foods, dietary supplements, and pharmaceuticals without sacrificing taste. D-Mannose is a biologically active monosaccharide that can be obtained from coffee residues through integrated processes involving delignification, degreasing, and saccharification steps or by (auto)hydrolysis [29,66]. Regarding sugar alcohols, sorbitol (E420) can be produced from corn and rice straw by hydrolysis of cellulose to glucose followed by its hydrogenation to sorbitol, or through a one-step synthesis from cellulose (with hydrolysis and hydrogenation in the same reactor) using Ru_2_P/C–SO_3_H as a catalyst [67]. Xylitol (E967), which has a sucrose-like sweetness but a lower glycemic index, can be produced from xylose-rich hemicellulosic hydrolysates derived from lignocellulosic waste (Figure 3), such as wheat bran and rice straw, using *Debaryomyces hansenii* and *Candida* spp. strains [68]. In addition to the initial hydrolysis, xylose purification makes the process challenging. More complex and time-consuming biotransformation processes allow the utilization of glucose, but it must initially be converted into D-arabitol by yeasts to be used by xylitol-producing strains such as *Gluconobacter* spp. [68]. Recombinant *E. coli* strains expressing key genes from *Gluconobacter* spp. have been developed to improve the bioconversion of D-arabitol into xylitol, with yields up to 0.87 g/g [68].

**Figure 4:**
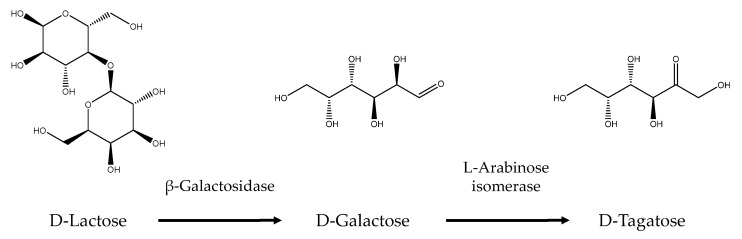
Upcycling of D-lactose from dairy waste into D-tagatose through a multi-enzyme cascade. The enzyme β-galactosidase converts D-lactose into D-galactose, and L-arabinose isomerase catalyzes the isomerization of D-galactose into D-tagatose.

On the other hand, rare sugars are a promising alternative to sugar alcohols in sugar-free products. These next-generation sugar substitutes can be produced using three types of enzymes: aldo–keto isomerases, carbohydrate epimerases, and oxidoreductases [69]. For example, the enzyme L-arabinose isomerase catalyzes the reversible isomerization of L-arabinose to L-ribulose and can also convert D-galactose to D-tagatose. Several microorganisms efficiently produce L-arabinose isomerase, facilitating the production of L-ribulose or D-tagatose [70]. As shown in Figure 4, D-tagatose can be produced from dairy waste and D-lactose through a multi-enzyme cascade [29]. D-tagatose is an isomer of fructose, about 90% sweeter than sucrose, and is a promising low-calorie sweetener (E963) with antidiabetic properties [71]. It can also be obtained from onion waste by enzymatic bioconversion using purified *Paenibacillus polymyxa* L-arabinose isomerase. This enzyme exhibits maximal activity at 30 °C and pH 7.5, producing 99 mg of D-tagatose per gram of onion waste with a conversion yield of 46.94% from D-galactose to D-tagatose [70]. This rare sugar can also be obtained from the macroalgae *Ulva* spp. and *Spirogyra* spp., yielding 1.84 mg/g and 4.12 mg/g of polysaccharide extract, respectively. This extraction method involved heating the algae biomass in water at 98 °C for 1 h, followed by precipitation with ethanol and drying to obtain crude polysaccharide extracts, which were then partially purified through hot water extraction and acetone precipitation [72].

Oligosaccharides such as GOS and XOS are prebiotic fibers with moderate sweetness that promote health benefits by inducing specific changes in the composition and/or activity of the gastrointestinal microbiota, as they are fermented by beneficial gut bacteria [73]. GOS are lactose-derived compounds containing β-linked galactose moieties and are synthesized using β-galactosidase as a biocatalyst through a transgalactosylation reaction, with lactose as the primary substrate. Immobilizing β-galactosidase in calcium alginate produces GOS with a purity of 28.7% (*w*/*w*) from 380 g/L lactose solution at pH 5.4 and 50 °C. Further purification can be achieved through fermentation with *Saccharomyces cerevisiae* and *Kluyveromyces lactis* entrapped in calcium alginate, with *S. cerevisiae* maintaining a purity above 37% for nineteen batches and *K. lactis* producing over 97% purity for two batches [74]. Despite the established β-galactosidase-based GOS synthesis methods, achieving high-purity, large-scale production remains a challenge for improving industrial yields. In turn, XOS can be produced from plant and microbial sources, with large-scale production from lignocellulose FLW biomass being crucial due to the abundance of xylan, a polysaccharide mainly composed of xylose residues, which serves as its precursor [73]. This presents an opportunity to meet future XOS demands sustainably, using FLW such as sugarcane bagasse, wheat and rice straw, and other crop by-products. Since hemicellulose is the biomass fraction that solubilizes most easily and single-step pretreatments to achieve high solubilization yields can be too severe, a high concentration of monomers can be obtained, impairing the purification stage [75]. As a result, two-step approaches combining pretreatment with enzymatic hydrolysis are recommended, with xylanases playing a key role in XOS production, generally yielding less undesired compounds, which simplifies purification [75]. However, XOS yield can vary significantly based on the type of FLW biomass and applied technology, and the lack of an efficient bioprocess for converting lignocellulosic xylan into XOS remains a major challenge for large-scale production.

## 4. Biotechnological and Societal Challenges

The biorefinery concept has evolved in recent years and focuses on multi-product cascade processes, emerging as the most promising for FLW biotransformation into food-grade products. However, to ensure commercial feasibility, major efforts are still needed to improve the efficiency and techno-economic performance of these biorefineries, production processes, and facilities. The main challenges and development needs are related to the following: (i) The selection of the most suitable feedstock for an effective production of specific metabolites and their best microbial utilization, together with the development of more eco-efficient pretreatments for recalcitrant and heterogeneous feedstocks. (ii) The bioreactor design, incorporating cutting-edge technologies for better monitoring and control of different process parameters and overcoming scale-up setbacks. (iii) The improvement and better control of the culture and fermentation/biotransformation conditions through the optimization of variables that affect the process (pH, temperature, moisture, agitation, etc.) [39] and, consequently, the yield and biomass utilization. (iv) The selection and genetic improvement of polyvalent microbial strains with superior metabolizing capacity. (v) The creation of easy-to-use, integrated fermentation and product recovery platforms, as well as more efficient separation and purification methods. (vi) The scale-up and techno-economic and life-cycle analysis of biorefineries. (vii) For certain products (e.g., peptides, which are easily disturbed by the food matrix and food processing conditions), stabilization techniques may be needed to allow their application in different food or nutraceutical matrices.

Food safety and consumer acceptance concerns may arise due to the potential presence of chemical and biological contaminants in FLW feedstock and the “waste” source of upcycled bio-based ingredients. Therefore, ensuring the safety of this feedstock is crucial when incorporating it into the food chain. However, there is limited research evaluating the presence of chemical and microbiological contaminants, such as pesticide residues, mycotoxins, pathogenic bacteria, and other organic and inorganic substances [76]. In this regard, efforts are underway to develop decontamination procedures and more sensitive analytical methods [77,78]. Despite these advances, specific legislation, monitoring, and surveillance are required to regulate upcycled products’ quality, safety, and suitability, ensuring consumer protection. Furthermore, the safety assessment of FLW from different sources requires greater attention from both researchers and industry to establish standards and protocols for efficient and safe valorization and upcycling. Downstream, targeted education and awareness initiatives are important for addressing consumer concerns about the safety and acceptability of upcycled products, particularly food items.

## 5. Conclusions

Food waste generation continues to rise worldwide, and given the modern world’s challenges to its prevention, its mass-scale valorization appears as a major step towards a more sustainable agri-food value chain, food security, circular bioeconomy, and climate change mitigation. This can be achieved through cutting-edge biotechnological biorefinery processes that allow the transformation of these renewable feedstocks into food-grade ingredients and specialty chemicals, such as organic acids, phenolic compounds, bioactive peptides, sweeteners, and prebiotics, among other valuable metabolites. These bio-based products emerge as natural alternatives to some synthetic or artificially obtained molecules currently used as additives and can be used to formulate clean-label foods, food supplements, nutraceuticals, and other consumer goods. However, increased investment, biotechnological advancement, and public awareness emerge as key drivers of progress that various stakeholders must work on to achieve multi-product cascade biorefining schemes, FLW decomposition to sub-zero level, and safe, high-quality upcycled products.

## Data Availability

No new data were created or analyzed in this study. Data sharing is not applicable to this article.
